# Chronic exposure to low-level lipopolysaccharide dampens influenza-mediated inflammatory response *via* A20 and PPAR network

**DOI:** 10.3389/fimmu.2023.1119473

**Published:** 2023-01-16

**Authors:** Yinuo Gu, Alan Chen-Yu Hsu, Xu Zuo, Xiaoping Guo, Zhengjie Zhou, Shengyu Jiang, Zhuoer Ouyang, Fang Wang

**Affiliations:** ^1^ Department of Pathogeny Biology, College of Basic Medical Sciences, Jilin University, Changchun, China; ^2^ Signature Research Program in Emerging Infectious Diseases, Duke - National University of Singapore (NUS) Graduate Medical School, Singapore, Singapore; ^3^ School of Medicine and Public Health, The University of Newcastle, Newcastle, NSW, Australia; ^4^ Viruses, Infections/Immunity, Vaccines and Asthma, Hunter Medical Research Institute, Newcastle, NSW, Australia

**Keywords:** A20 (TNFAIP3), IAV, PPAR, NF-κB, NLRP3 inflammasome

## Abstract

Influenza A virus (IAV) infection leads to severe inflammation, and while epithelial-driven inflammatory responses occur *via* activation of NF-κB, the factors that modulate inflammation, particularly the negative regulators are less well-defined. In this study we show that A20 is a crucial molecular switch that dampens IAV-induced inflammatory responses. Chronic exposure to low-dose LPS environment can restrict this excessive inflammation. The mechanisms that this environment provides to suppress inflammation remain elusive. Here, our evidences show that chronic exposure to low-dose LPS suppressed IAV infection or LPS stimulation-induced inflammation *in vitro* and *in vivo*. Chronic low-dose LPS environment increases A20 expression, which in turn positively regulates PPAR-α and -γ, thus dampens the NF-κB signaling pathway and NLRP3 inflammasome activation. Knockout of A20 abolished the inhibitory effect on inflammation. Thus, A20 and its induced PPAR-α and -γ play a key role in suppressing excessive inflammatory responses in the chronic low-dose LPS environment.

## Introduction

IAV frequently causes severe infection with heightened inflammation that drives disease pathogenesis. While inflammation is essential in virus clearance, excessive inflammatory responses cause epithelial tissue destruction, acute lung injury and pneumonia (1, 2). Upon infection in airway epithelial cells, the primary site of IAV infection, IAV viral RNAs are recognized by the host pattern recognition receptors including retinoic acid inducible gene (RIG)-I and toll-like receptor (TLR)3. RIG-I binding to viral RNAs leads to activation of interferon regulatory factor (IRF)3 that facilitate the production of antiviral cytokines type I interferon (IFN). TLR3 promotes the activation of nuclear factor kappa-lightchain-enhancer of activated B cells (NF-κB), leading to the induction of pro-inflammatory cytokines such as interleukin (IL)-6 and tumour-necrosis-factor (TNF)α. Upon infection or stimulation, NLRP3, ASC, and pro-caspase-1 assembles to form an inflammasome complex, which activates pro-caspase-1 by cleavage. IL-1β is a potent inflammatory cytokine that is produced as a pro-protein that is then cleaved by caspase-1 (3).

It has been suggested that the host inflammatory response to IAV is more likely to have serious consequences than viral load (1).

The mechanisms that drive NF-κB pathway are well characterized, however the negative control factors that dampen NF-κB activation is less well studied. A recent study showed that chronic exposure of low-dose lipopolysaccharide (LPS) protected against the development of allergy and asthma (4, 5).

The molecular mechanisms underpinning this observation is unclear. We recently showed that A20 (also known as TNFα-induced protein 3) was crucial in limiting IAV-mediated NF-κB activation and IAV diseases *in vitro* and *in vivo*. The mechanisms by which low-grade LPS and A20 protect the host from heightened inflammation is unknown. In this study we determined that human airway epithelial cells and mice that were chronically exposed to low-dose LPS reduced subsequent IAV- and high-dose LPS-mediated NF-κB and NLRP3 activation, and this low-grade LPS tolerance was mediated by increased A20 and PPAR-α and PPAR-γ expression, the latter of which were also negative regulators of NF-κB.

## Materials and methods

### Cell culture and IAV

Human lung adenocarcinoma epithelial (A549) cells and Madin-Darby canine kidney (MDCK) cells were purchased from the Chinese Academy of Sciences Cell Bank (Shanghai, China). A549 cells were cultured in Ham’s F-12 K medium supplemented with 10% fetal bovine serum (FBS). MDCK cells were cultured in DMEM medium supplemented with 10% FBS. All cells were cultured with 5% CO_2_ at 37°C.

H1N1 influenza virus, A/Fort Monmouth/1/1947 virus (FM1), was propagated in the allantoic cavities of 9-11 day-old embryonated specific pathogen-free chicken eggs cultured at 37°C. After 48 h allantoic fluids of infected eggs were collected. Various dilutions of allantoic fluids were incubated with MDCK cells to determine the 50% tissue culture infectious dose (TCID_50_) calculated by the Reed-Muench method. TCID_50_ is 10^-4.1^. To determine the 50% lethal dose (LD_50_), the allantoic fluids were serially 10-fold diluted. The number of deaths and survivals in each dilution was used to calculate the LD_50_ by the Reed–Muench method. Based on the titrations, 1 LD_50_ of the allantoic fluids was determined to be 10^-5.5^.

### Animals and animal experiments

Female BALB/c mice (6–8 weeks old) were purchased from the Yisi Laboratory Animal Technology Co., Ltd. (Changchun, China), and maintained in micro-isolator cages under specific pathogen-free conditions. The experimental manipulation of the mice was undertaken in accordance with the National Institute of Health Guide for the Care and Use of Laboratory Animals, with the approval of the Scientific Investigation Board of Science & Technology of Jilin Province, China. All mice received human care in compliance with the 2011 Guide for the Care and Use of Laboratory Animals published by the National Institutes of Health. The mouse experiments were approved by the ethics committee of The College of Basic Medical Sciences of Jilin University with the number 2022-467.

We intranasally administered with 100 ng LPS for chronic-LPS^lo^ group or PBS for control group every other day. After 2 weeks, both groups of mice were treated with non-infectious allantoic fluid, H1N1 (10 LD_50_), and high-dose (10 µg/mL) LPS. Both H1N1 and high-dose LPS were inoculated intranasally after mice were anesthetized with 10% chloral hydrate intraperitoneally. Mice were sacrificed 24 h post infection points for sampling.

### Histological analysis and haematoxykin & eosin staining

The lungs of the infected mice were fixed in 4% (weight/vol.) paraformaldehyde, embedded into paraffin and cut into 4-μm-thick sections. The sections were stained with haematoxylin & eosin, and observed under the microscope. Pathological scores of the lungs were determined based on the criteria: 0, normal lung tissue structure with no inflammatory cell infiltration; 1, normal alveolar structure, mild lung injury (< 25% of the lung) with little inflammatory cell infiltration; 2, alveolar collapse, moderate injury (25– 50% of the lung) with some inflammatory cell infiltration; 3, no alveolar structure, severe injury (> 50% of the lung) with massive inflammatory cell infiltration.

### Chronic low-dose LPS stimulation, H1N1 infection and high-dose LPS stimulation in A549 cells

In the chronic-LPS^lo^ group cells were exposed to low-dose LPS (1 ng/mL) every other day for 4 weeks by adding low-dose LPS to the culture the day after each passage, the control group cells exposed with PBS. After four weeks, cells in both groups were treated with media, H1N1, and high-dose (1µg/mL) LPS. A549 was inoculated with 10^2^ TCID_50_/mL H1N1 for 2h, after which the inoculum was removed and fresh serum-free media was added and incubated at 37°C/5% CO_2_ for 24h.

### Sample preparation and analysis of tandem mass tags labeled quantitative proteomics

A549 cells in control+H1N1 group, chronic-LPS^lo^+H1N1 group, control+acute-LPS^hi^ and chronic-LPS^lo^+acute-LPS^hi^ group were harvested for LC-MS analyses. Proteins were precipitated with 25mM DL-dithiothreitol, iodoacetamide, precooled acetone. After pellets we collected by centrifugation, the protein pellets were redissolved with enzymolysis diluent, followed by lyophilization and TMT labeling. Proteomic analysis was performed using a Q-Exactive mass spectrometer (Thermo, USA) after reversed-phase (RP) separation. Proteome Discover 2.4 (Thermo, USA) was used to process LC-MS/MS data, and credible proteins were screened using Score Sequest HT > 0 and unique peptide ≥ 1. Significant differences in proteins between the two groups were determined by performing Student’s t-test. Fold changes less than 0.67 or more than 1.5 and p-values less than 0.05 were considered significant. Annotation information of each identified protein was extracted using Uniprot database. Pathway enrichment analysis of differential proteins was performed based on the Kyoto Encyclopedia of Genes and Genomes (KEGG) database.

### Generation of A20 KO cells by CRISPR-Cas9

CRISPR−Cas9 system was used to knock out A20 expression in A549 cells. Briefly, two synthetic guide RNA (sgRNA) sequence targeting exon 3 of the A20 gene were designed online (https://www.benchling.com/crispr/) and then cloned into the CRISPR/Cas9 48138-puro vector. The sgRNA sequences were: sgRNA#1: AGGGGTACCCTATGCCCACC; sgRNA#2: CAGCCCTACTGCTATTCTAG. A20 cell clones were selected using green fluorescent protein (GFP) expression and restriction fragment length polymorphism.

### Cytokine analysis

TNF-α, IL-6, IL-1β in the supernatant and mouse serum and IFN-α, IFN-β, IFN-γ and IL-18 in mouse alveolar lavage fluid were measured using ELISA kits (Lianke, Hangzhou, China), according to the manufacturer’s protocol.

### Western blotting

Cells and lung tissues were lysed in RIPA buffer, and protein concentration determined by BCA protein assay kit (Beyotime, Shanghai, China). Denatured protein samples (20μg) were separated by 8% or 15% SDS-PAGE and transferred onto PVDF membranes. The membranes were blocked in 5% nonfat milk for 3 h at room temperature, and probed with specific primary antibodies (1:1000) against p65 (ab16502), p-p65 (ab76302), A20 (ab92324), NLRP3 (ab214185), pro-caspase-1 (ab179515), caspase-1 (ab179515), PPAR-α (ab61182), PPAR-γ (ab209350) and β-actin (ab8227) (Abcam, Cambridge, UK) overnight at 4°C. Goat-derived anti-rabbit HRP conjugated antibody (1:2000) were used as secondary antibody for 1 h. Membranes were developed and analyzed using the Super Signal Chemiluminescent Substrate kit (Thermo Scientific, Rockford, USA) and ImageJ software.

### mRNA analysis

Total RNAs from A549 cells and lung tissues were extracted using Trizol (Thermo Scientific, Rockford, USA). RNA was reverse-transcribed to cDNA using first strand cDNA synthesis kit (Thermo Scientific, Rockford, USA). qPCR assays were performed with SYBR Green PCR Master Mix (TaKaRa, Japan) and a Fast qPCR System (Applied Biosystems, USA). GAPDH was used as the internal reference. The relative mRNA level was calculated by 2^−ΔΔCt^ method. The primers used were GAPDH (human), F; TCTTCTTTTGCGTCGCCAG, R; AGCCCCAGCCTTCTCCA, IL-1β (human), F; CTGTCCTGCGTGTTGAAAGA, R; TTGGGTAATTTTTGGGATCTACA, IL-6 (human), F; CACTGGTCTTTTGGAGTTTGAG, R; GGACTTTTGTACTCATCTGCAC, TNF-α (human), F; AGCCCTGGTATGAGCCCATCTATC, R; TCCCAAAGTAGACCTGCCCAGAC, TNFAIP3 (human), F; TGCACACTGTGTTTCATCGAG, R; ACGCTGTGGGACTGACTTTC, GAPDH (mouse), F; AGGTCGGTGTGAACGGATTTG, R; TGTAGACCATGTAGTTGAGGTCA, IL-1β (mouse), F; CACTACAGGCTCCGAGATGAACAAC, R; TGTCGTTGCTTGGTTCTCCTTGTAC, IL-6 (mouse), F; CTTCTTGGGACTGATGCTGGTGAC, R; TCTGTTGGGAGTGGTATCCTCTGTG, TNF-α (mouse), F; CGCTCTTCTGTCTACTGAACTTCGG, R; GTGGTTTGTGAGTGTGAGGGTCTG, TNFAIP3, F; TCCTCAGGCTTTGTATTTGAGC, R; TGTGTATCGGTGCATGGTTTTA.

### Immunofluorescence

A549 cells were cultured in 24 well plate. The cells were fixed in 4% paraformaldehyde for 10 min and then permeabilized with 0.02% Triton X for 5 min. The cells were blocked with goat serum for 60 min. The cells were incubated with primary antibodies against p65 overnight at 4°C. The cells were incubated with the secondary antibodies for 60 min and then stained by DAPI. Fluorescent images were captured with an immunofluorescence microscope (IX71, Olympus, Japan).

### Statistical analysis

Statistical analysis was performed using Prism software (GraphPad). Student’s t test or One-way analysis of variance (ANOVA), followed by Tukey’s *post hoc* analysis was used for calculation of statistical differences. p values <0.05 were considered statistically significant.

## Results

### Chronic low-dose LPS stimulation ameliorates symptoms and prolongs survival in H1N1-infected mice

**Figure 1:**
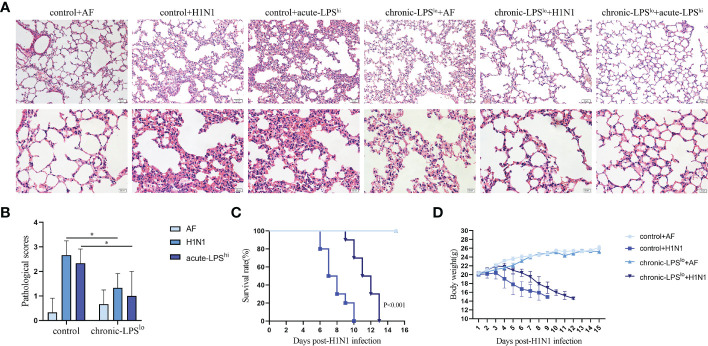
The effect of chronic low-dose LPS stimulation on lethal IAV infected mice. **(A)** Female BALB/c mice (n = 3 mice/group) were intranasally infected with 10 LD_50_ H1N1 influenza virus or an equivalent dilution of non-infectious allantoicfluid (AF). The lungs were isolated and sectioned for hematoxylin & eosin staining. **(B)** Pathological scores of the stained sections. **(C)** Survival data of female BALB/c mice (n = 10 mice/group) after intranasal infection with 10 LD_50_ H1N1. **(D)** Body weights of female BALB/c mice (n = 10 mice/group) after intranasal infection with 10 LD_50_ H1N1. *p< 0.05.

Chronic-LPS^lo^ stimulation has been well studied in childhood asthma and farm environments protect children from asthma through exposure to house dust mites and LPS (4, 5). To explore whether chronic-LPS^lo^ stimulation is protective against H1N1 or high-dose LPS stimulation *in vivo*, we exposed mice to low-dose LPS or control PBS every other day for two weeks prior to H1N1 infection and high-dose LPS stimulation. We examined the lung histological changes of mice in each group, both H1N1 and acute-LPS^hi^ resulted in severe lung injury, diffuse swelling, alveolar cavity collapse, alveolar thickening, and severe infiltration of inflammatory cells (**Figure 1A**). Chronic-LPS^lo^ stimulation significantly improved lung injury as assessed by pathology scores compared with control+H1N1 and control+acute-LPS^hi^ groups (**Figure 1B**).

We recorded symptoms and body weight changes in H1N1-infected mice daily. The results showed that on day 2 after intranasal administration of H1N1, the mice began to show signs of decreased activity, loss of appetite, hunched back and ruffled fur. By day 5, mice may experience dyspnea and respiratory distress. Mice in the control group started dying on day 6 post-infection and all died within 10 days post-infection (**Figure 1C**). The mice gradually lost weight starting on the third day after infection until death (**Figure 1D**).

Chronic-LPS^lo^ stimulation did not induce significant pathological changes in the lungs of mice, nor cause weight loss in mice, and ameliorated the aforementioned disease symptoms and delayed weight loss and prolonged survival.

### Chronic low-dose LPS stimulation inhibits the activation of NF-κB signaling pathway and NLRP3 inflammasome

**Figure 2:**
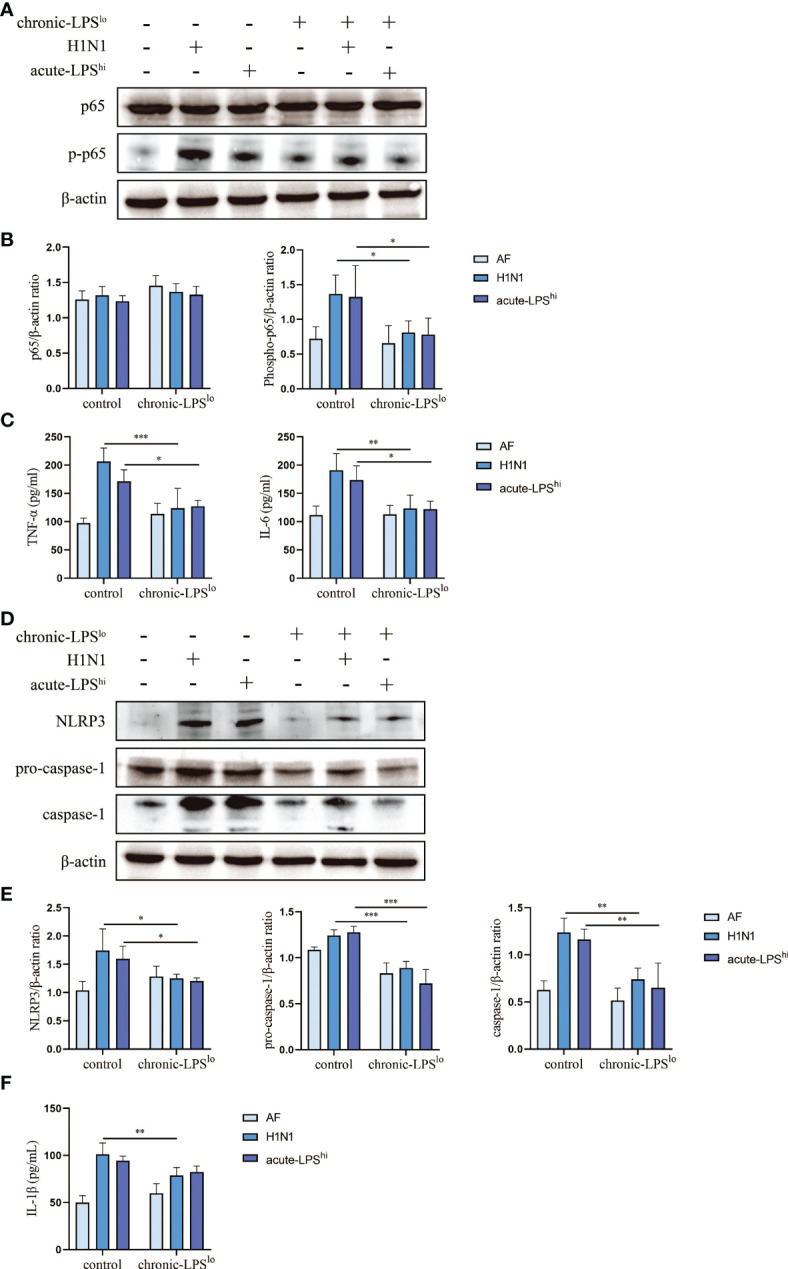
Effect of chronic low-dose LPS stimulation on NF-κB signaling pathway and NLRP3 inflammasome in high dose LPS-stimulated or H1N1-infected mice. **(A, B)** Relative protein levels of p65 and p-p65 in lung tissues were detected by western blot. **(C)** TNF-α and IL-6 in mice sera were measured by ELISA. **(D, E)** Relative protein levels of NLRP3, pro-caspase-1 and caspase-1 in lung tissues were detected by western blot. **(F)** IL-1β in mice sera were detected by ELISA. Data are mean ± SD, n = 3. *p< 0.05, **p< 0.01, ***p< 0.001.

The activation of NF-κВ pathway and NLRP3 inflammasome during infection leads to upregulation of pro-inflammatory cytokines expression. To assess whether severe cytokine storms were generated during H1N1 infection or high-dose LPS stimulation and further clarify the mechanism by which chronic-LPS^lo^ stimulation inhibits inflammation, we analyzed the effects of chronic-LPS^lo^ stimulation on the regulation of NF-κВ pathway and NLRP3 inflammasome. In H1N1-infected or high-dose LPS-stimulated mice, chronic-LPS^lo^ stimulation significantly decreased p65 phosphorylation (**Figures 2A, B**). We examined the production of TNF-α and IL-6 downstream of the NF-κВ pathway in mice lung tissues and sera. Compared with the control+AF group, H1N1 infection significantly increased the mRNA and protein levels of TNF-α and IL-6 (**Figures 2C**, **S1 A, B**).

Both H1N1 infection and acute-LPS^hi^ stimulation independently induced NLRP3 inflammasome activation, including increased NLRP3 and pro-caspase-1 protein expression and activated caspase-1 production. Chronic-LPS^lo^ stimulation decreased NLRP3, pro-caspase-1 and caspase-1 at protein levels (**Figures 2D, E**). We examined the production of IL-1β downstream of the NLRP3 inflammasome in mice lung tissues and sera. Chronic-LPS^lo^ stimulation significantly reduced the increase in the expression of IL-1β (**Figures 2F**, **S1 C**).

We also examined IFN-α, IFN-β, IFN-γ and IL-18 production in mouse alveolar lavage fluid. Chronic-LPS^lo^ stimulation significantly reduced the increase in IFN-α and IFN-β expression, but had no significant effect on IFN-γ and IL-18 (**Figure S2**).

Our results showed that chronic-LPS^lo^ stimulation inhibited H1N1 or acute LPS^hi^ induced activation of NLRP3 inflammasome and NF-κB and production of pro-inflammatory cytokines.

### Chronic low-dose LPS stimulation upregulates A20 expression

**Figure 3:**
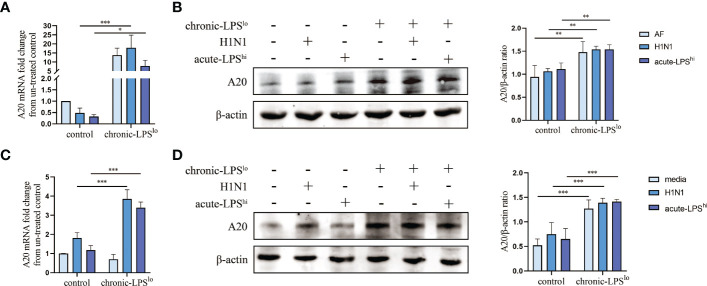
Effect of chronic low-dose LPS stimulation on A20. **(A)** A20 mRNA in lung tissues was detected by qPCR. **(B)** Relative protein levels of A20 in lung tissues were detected by western blot. **(C)** A20 mRNA in A549 cells was detected by qPCR. **(D)** Relative protein levels of A20 in A549 cells were detected by western blot. Data are mean ± SD, n = 3. *p< 0.05, **p< 0.01, ***p< 0.001.

A20 as a key protein in the protection mediated by chronic exposure to low-dose LPS environment is also a major determinant of progression and prognosis in several other inflammatory diseases (6–9). To observe the effects of chronic-LPS^lo^ stimulation on human airway epithelial cells, we exposed A549 cells for 4 weeks to a low-dose of LPS (1ng/mL) or to control PBS before acute H1N1 infection or acute high-dose LPS stimulation. We next assessed the expression of A20 and showed that the mRNA and protein levels of A20 were significantly increased in the chronic-LPS^lo^ group than in the control group *in vivo* (**Figures 3A, B**) and *in vitro* (**Figures 3C, D**).

### A20 knockout abolishes the anti-inflammatory effects by chronic low-dose LPS stimulation

**Figure 4:**
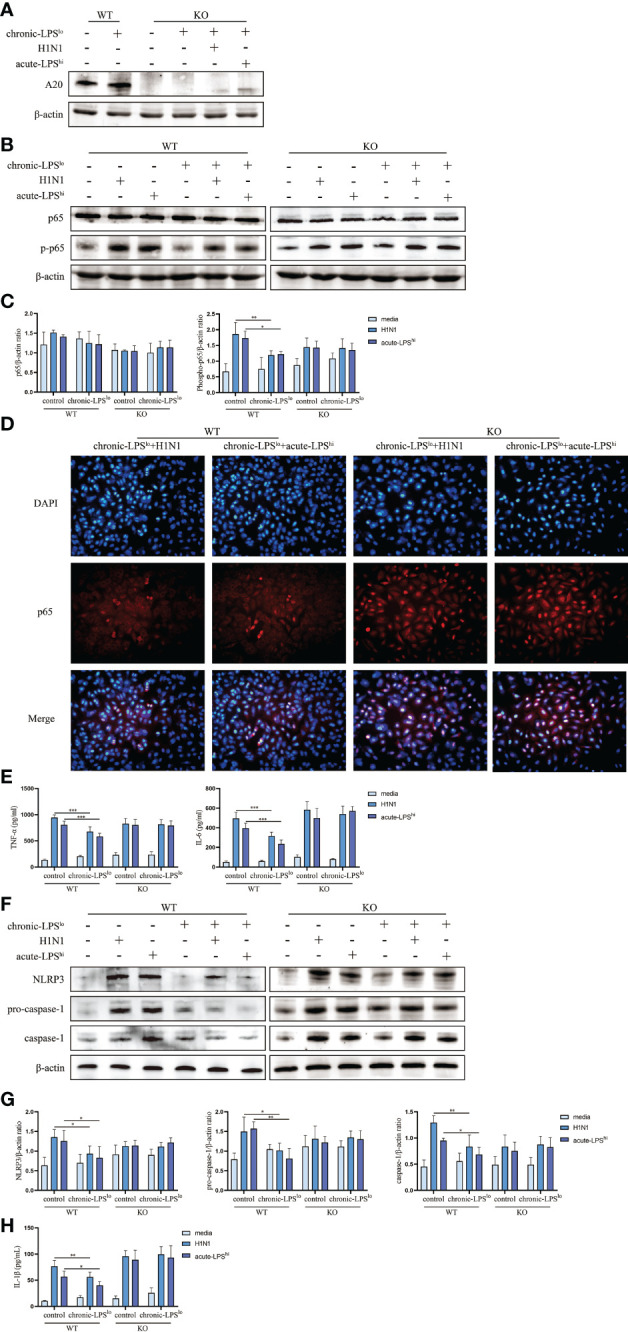
Effect of chronic low-dose LPS stimulation on NF-κB signaling pathway and NLRP3 inflammasome in high dose LPS-stimulated or H1N1-infected A549^WT^ and A549^A20-KO^ cells. **(A)** Relative protein levels of A20 in A549^A20-KO^ cells were detected by western blot. **(B, C)** Relative protein levels of p65 and p-p65 in A549^WT^ cells and A549^A20-KO^ cells were detected by western blot. **(D)** Immunofluorescent images of the localization of p65 in A549^WT^ and A549^A20-KO^ cells. **(E)** TNF-α and IL-6 in A549^WT^ and A549^A20-KO^ cells culture supernatants were measured by ELISA. **(F, G)** Relative protein levels of NLRP3, pro-caspase-1 and caspase-1 in A549^WT^ and A549^A20-KO^ cells were detected by western blot. **(H)** IL-1β in A549^WT^ and A549^A20-KO^ cells culture supernatants were measured by ELISA. Data are mean ± SD, n = 3. *p< 0.05, **p< 0.01.

To further assess the roles of A20 in dampening H1N1 and acute-LPS^hi^-mediated inflammatory responses, we generated A20-deficient A549 cells (A549^A20-KO^) using CRISPR-Cas9 gene editing, and showed a complete lack of A20 protein expression (**Figure 4A**). A549^A20-KO^ cells proliferated at similar rate as wild-type cells (A549^WT^) and did not show significant morphological differences.

We measured the levels of activity NF-κB by assessing the levels of phosphorylated p65 (p-p65). H1N1 infection or LPS stimulation significantly increased the activation of p-p65 in both control H1N1 and control acute-LPS^hi^ groups. The inhibition of chronic LPS^lo^ stimulation to nuclear translocation of p65 in H1N1-infected and high-dose LPS-stimulated A549^A20-KO^ cells was lost (**Figures 4B–D**). There was also no significant difference in the production of TNF-α and IL-6 between the control group and the chronic-LPS^lo^ group (**Figures 4E**, **S3 A, B**). These results indicated that the inhibitions of chronic-LPS^lo^ stimulation on NF-κB signaling pathway in H1N1-infected and high-dose LPS-stimulated A549 cells were A20 dependent.

Next, we detected the effect of chronic-LPS^lo^ stimulation on NLRP3 inflammasome after knockout of A20. Knockout of A20 made the inhibition of NLRP3 signaling pathway by chronic low-dose LPS stimulation lost (**Figures 4F, G**). The inhibitory effect of chronic-LPS^lo^ stimulation on the production of IL-1β also disappeared (**Figures 4H**, **S3 C**). These results indicated that the inhibitions of chronic-LPS^lo^ stimulation on NLRP3 inflammasome in H1N1-infected and high-dose LPS-stimulated A549 cells were A20 dependent.

### Chronic low-dose LPS stimulation inhibits H1N1-infected or high-dose LPS-stimulated inflammation *via* PPAR

**Figure 5:**
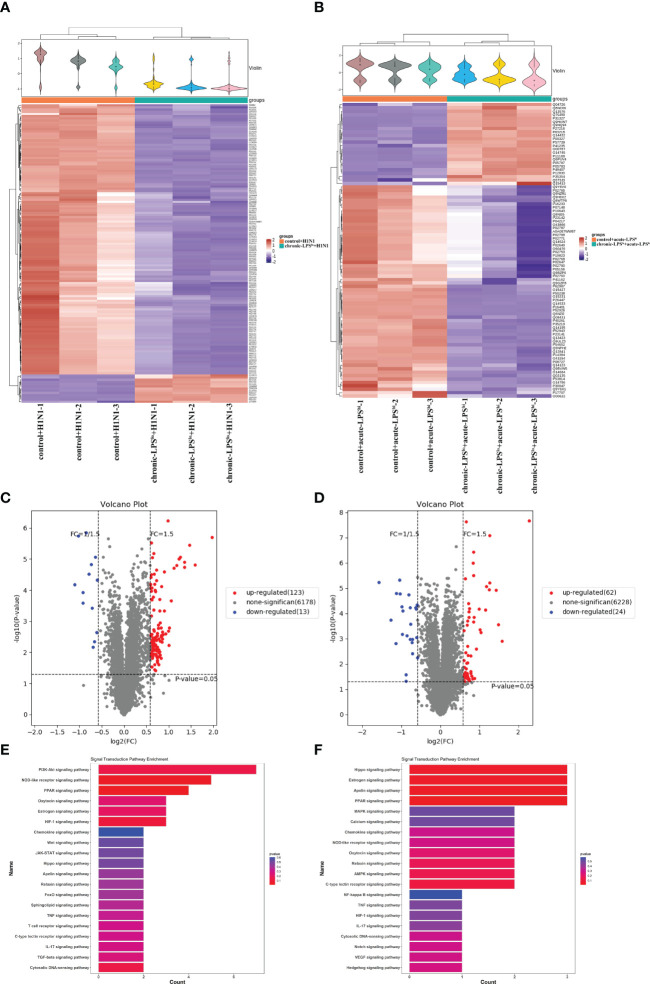
Inhibition of inflammation by chronic low-dose LPS stimulation is related to PPAR. **(A)** Hierarchical clustering analysis heat map in control+H1N1 group compared with chronic-LPS^lo^+H1N1 group. **(B)** Hierarchical clustering analysis heat map in control+acute-LPS^hi^ group compared with chronic-LPS^lo^+acute-LPS^hi^ group. **(C)** The differentially expressed proteins analyzed by volcano plots in control+H1N1 group compared with chronic-LPS^lo^+H1N1 group (vertical dotted lines, fold change > 1.5-fold; horizontal dotted line, p < 0.05). **(D)** The differentially expressed proteins analyzed by volcano plots in control+acute-LPS^hi^ group compared with chronic-LPS^lo^+acute-LPS^hi^ group (vertical dotted lines, fold change > 1.5-fold; horizontal dotted line, p < 0.05). **(E)** Top 20 of signaling transduction of KEGG pathway enrichment analysis in control+H1N1 group compared with chronic-LPS^lo^+H1N1 group. **(F)** Top 20 of signaling transduction of KEGG pathway enrichment analysis in control+acute-LPS^hi^ group compared with chronic-LPS^lo^+acute-LPS^hi^ group.

To assess the global changes induced by chronic-LPS^lo^ stimulation at the protein level, we subjected the cells from control+H1N1 group, chronic-LPS^lo^+H1N1 group, control+acute-LPS^hi^ and chronic-LPS^lo^+acute-LPS^hi^ group to TMT-labeled quantitative proteomics. We identified 6,178 quantitative proteins in the control+H1N1 group and chronic-LPS^lo^+H1N1 group, of which 136 proteins had significantly increased or decreased expression. We identified 6,228 quantified proteins in control+acute-LPS^hi^ and chronic-LPS^lo^+acute-LPS^hi^ group, of which 86 proteins had significantly increased or decreased expression. Cluster analysis and heat map showed differentially expressed proteins control+H1N1 group compared with chronic-LPS^lo^+H1N1 group (**Figure 5A**) and control+acute-LPS^hi^ group compared with chronic-LPS^lo^+acute-LPS^hi^ group (**Figure 5B**).

The volcano plot showed that 123 proteins that were increased and 13 proteins that were decreased in the 136 differential proteins in chronic-LPS^lo^+H1N1 group compared with control+H1N1 group (**Figure 5C**). 62 proteins were increased and 24 proteins were decreased in the 86 differential proteins in chronic-LPS^lo^+acute-LPS^hi^ group compared with control+acute-LPS^hi^ group (**Figure 5D**).

KEGG pathway analysis showed the top 20 pathways that are well characterized in both H1N1 infection and LPS stimulation, including the PPAR, NOD-like receptor, NF-κB, TNF and chemokine signaling pathways. Interestingly we found that PPAR was highly enriched in chronic-LPS^lo^+H1N1 group (**Figure 5E**) and chronic-LPS^lo^+acute-LPS^hi^ group (**Figure 5F**).

### The increase of PPAR expression induced by chronic low-dose LPS stimulation is regulated by A20

Both PPAR-α and PPAR-γ inhibit NF-κB activation like A20 and PPAR-α has been reported to be positively regulated by A20 (10–12). In addition, PPAR-α/γ also exerts anti-inflammatory effects during influenza infection (13, 14). Despite the inflammatory signaling pathway is well characterized, whether PPAR-α/γ is involved in the mechanism by which A20 suppresses the excessive inflammatory response of IAV remains unclear.

**Figure 6:**
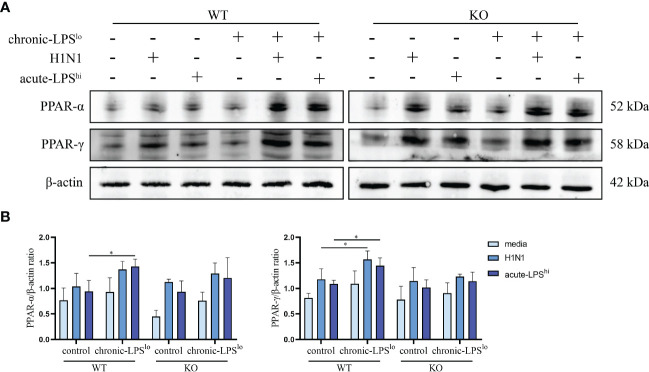
Effect of chronic low-dose LPS stimulation on PPAR-α and -γ. **(A, B)** Relative protein levels of PPAR-α and -γ in A549^WT^ and A549^A20-KO^ cells were detected by western blot. Data are mean ± SD, n = 3. *p< 0.05.

To explore whether PPAR plays a key role in the inhibitory effect of chronic-LPS^lo^ stimulation on inflammation, we measured the protein levels of PPAR-α and -γ in A549^WT^ cells and A549^A20-KO^ cells. Consistent with the proteomics, western blot analysis confirmed the increased expression of PPAR-α and -γ in chronic-LPS^lo^ groups. Furthermore, this elevated PPAR-α and -γ expression induced in chronic low-dose LPS stimulation was abolished in A549^A20-KO^ cells (**Figures 6A, B**), indicating that PPAR-α and -γ expression was regulated by A20, and loss of which resulted in exaggerated inflammatory responses by H1N1 or high-dose LPS.

## Discussion

**Figure 7:**
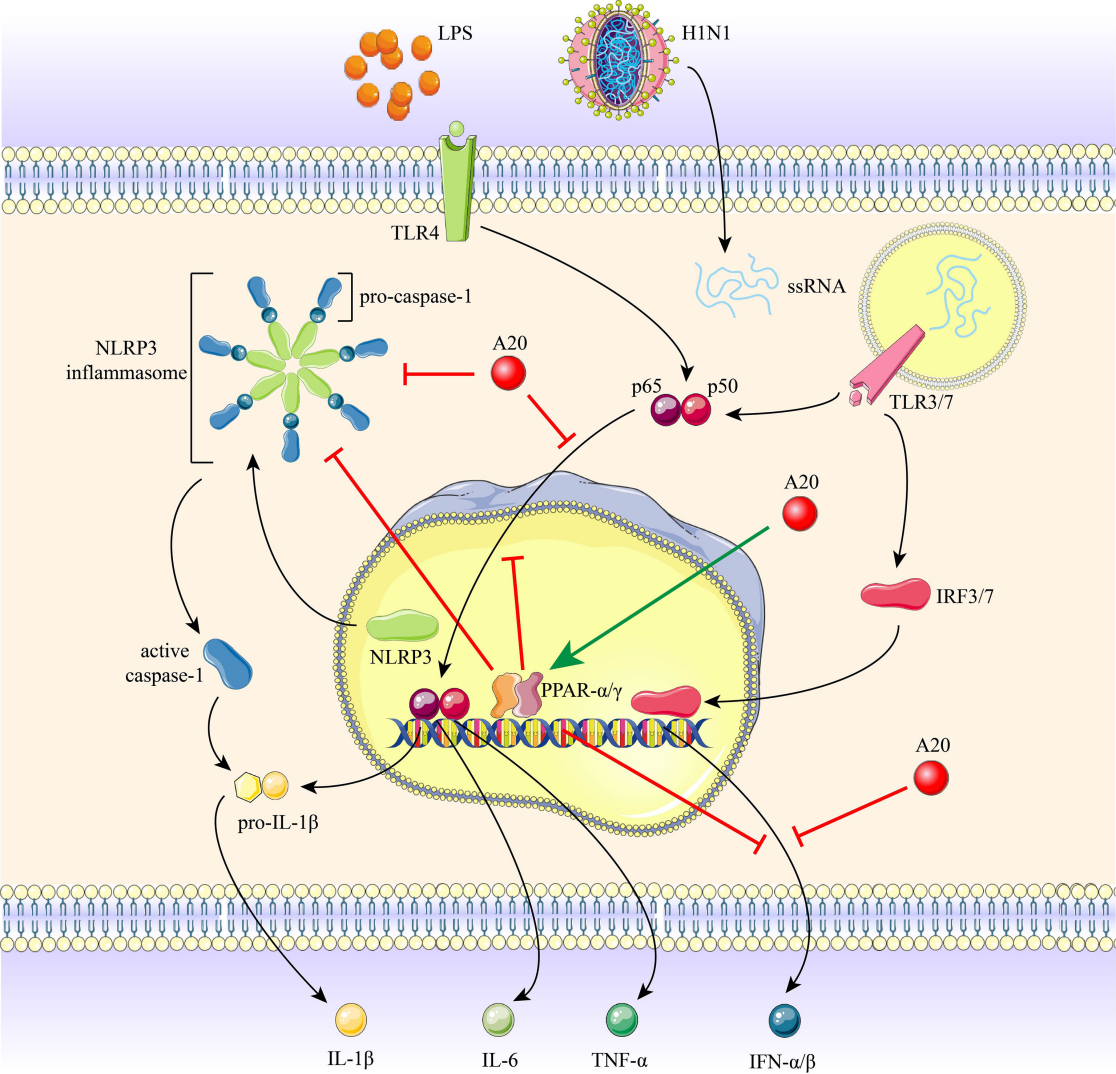
The role of A20 and PPAR-α and -γ in H1N1 infection and LPS stimulation induced inflammation. Recognition of H1N1 ssRNA or LPS by TLRs initiates the activation of NF-κB and NLRP3 inflammasome, promoting the production of downstream pro-inflammatory cytokines. Chronic low-dose LPS environment induced a marked increase in A20 expression, resulting in the restriction of NLRP3 inflammasome, caspase-1 cleavage, nuclear translocation of p65 subunit and IFNs expression. A20 increases PPAR-α and -γ expression, and PPAR-α and -γ also inhibit the above-mentioned inflammatory pathway.

Here, we demonstrate that H1N1 infection and high-dose LPS stimulation induce inflammation; however, the inflammatory response, NF-κB and NLRP3 activation were inhibited in chronic low-dose LPS environment. We show that A20 is a negative regulator of NF-κB and NLRP3-mediated inflammation and that A20 gene and protein levels are upregulated in a chronic low-dose LPS environment. Impaired inflammatory response in the chronic low-dose LPS environment was attributed to increased A20 and PPAR-α and -γ expression. Elevated A20 levels increased PPAR expression, leading to dampen of NF-κB and NLRP3 activity and inflammation. A20 knockout resulted in loss of chronic low-dose LPS-mediated suppression of inflammation. Thus, chronic low-dose LPS environment increases A20 and PPAR-α and -γ expression, in turn inhibits NF-κB and NLRP3 induced inflammation following IAV infection or high-dose LPS stimulation (**Figure 7**).

H1N1 causes serious health problems worldwide as a major infectious pathogen (15, 16). IAV infections cause severe airway inflammation and cytokine storms that lead to high morbidity and mortality (17, 18). Despite recent progress in the fight against IAV, control of inflammation remains a major challenge for severely ill patients (19–21). There are no effective treatments for the excessive inflammation and severe consequences in IAV, and the mechanisms of these events are poorly understood. Significant differences in influenza prevalence between metropolitan and rural areas suggest that environmental exposures may impact influenza severity (22). Possibly metropolitan cities have high hygiene standards, whereas people from small towns are protected because of continuous exposure to LPS or other microbial components (23, 24). Modulators targeting autoimmunity as preventive strategies such as chronic exposure to low-dose LPS environment is emerging as a promising strategy for a variety of inflammatory diseases, including IAV (4, 5).

A20 as a key protein in the protection mediated by chronic exposure to low-dose LPS environment is also a major determinant of progression and prognosis in several other inflammatory diseases (6–9). Environmental protection and A20 are well studied in childhood asthma, and farm environments protect children from asthma by increasing A20 expression (4, 5). Interestingly, A20 has been shown to inhibit NF-κB signaling pathway and NLRP3 inflammasome activation as well as autophagy, thereby preventing pulmonary fibrosis and arthritis (25–29). Here, we show that environment-mediated protection of A20 suppresses IAV or LPS induced inflammation and report new evidence for the molecular mechanisms that may be involved.

Here, we found that chronic-LPS^lo^ environment-mediated increases in A20 expression in cells significantly suppressed NF-κB signaling pathway activity and pro-inflammatory cytokine production to suppress IAV-induced hyper inflammation. Similar findings were reported in myeloid cells and lung epithelial cells (30, 31). Worth mentioning as the first line of defense against IAV infection, cytokines are a double-edged sword. Hyper induction of pro-inflammatory cytokine production also known as ‘cytokine storm’, it correlated directly with magnified inflammation and an unfavorable prognosis of IAV (18, 32, 33). Although cytokines play an important role in the antiviral response, if cytokines form a cytokine storm will hinder anti-virus immunity (34–36). Therefore, the exact role of A20 during viral infection requires further study. We could not rule out that other factors may also influence the relationship between A20 expression and IAV prognosis.

In addition to its key role in regulating NF-κB, several lines of evidence suggest that A20 regulates NLRP3 activation (37–39). For example, excessive Nlrp3 inflammasome activation drives arthritis pathogenesis in A20 knockout mice due to A20 putting a brake on Nlrp3 inflammasome activation by reducing LPS-induced Nlrp3 expression levels (29). Here, we consistently demonstrate that environment-mediated increases in A20 expression exhibit NLRP3 inflammasome suppression and IL-1beta expression downregulation upon infection with IAV or LPS stimulation, which is consistent with previous reports. Yet, we also explicitly show that A20 knockout cells no longer have the protection mediated by the chronic low-dose LPS environment. Our results combined with previous studies highlight the importance of increased A20 expression through the environment as a potential new therapeutic option for reducing IAV-mediated inflammation and cytokine storm.

We also found that PPAR-α and -γ plays an essential role in the protection of environment-mediated A20 elevation against inflammation, and PPAR-α and -γ are positively regulated by A20 and exert a similar inhibitory effect on inflammation as A20. Studies have shown that A20 protects against restenosis after carotid artery injury in rats through PPAR-α inhibition of NF-κB pathway activation (10). In addition, A20 can protect mice from lethal hepatic ischemia-reperfusion injury by increasing PPAR-α expression and inhibiting NF-κB activation (40). Unlike the tight link between PPAR-alpha and NF-κB, PPAR-gamma mainly inhibits NLRP3 inflammasome (41–44). Considering excessive inflammation is tied to IAV related mortality, PPAR-α/γ has been considered as a therapeutic target to limit such harmful inflammation. In a recent study, IAV infection was shown to reduce PPAR-γ mRNA levels in mouse alveolar macrophages (45). In addition, significant activation of PPAR-α and -γ by drugs can protect mice from IAV infection and pneumonia (14). It is crucial that cytokine production is finely tuned during anti-viral responses, in which it provides optimal protection while avoiding unwanted inflammation and tissue damage (46, 47). In this study the role of PPAR-α and -γ in environmental-mediated protection of chronic low-dose LPS the positive regulation of PPAR-gamma by A20 were explored for the first time. Whether the balance between A20 and inflammation is dependent on PPAR-α/γ, or whether there is crosstalk between A20 and PPAR-α/γ remains unclear; additional studies are required to resolve these points.

Collectively, our results suggest that inflammatory responses stimulated by H1N1 infection or high-dose LPS *in vitro* and *in vivo* were ameliorated in a chronic low-dose LPS environment. Increased levels of PPAR-α and -γ by A20 inhibits NF-κB and NLRP3 inflammasome activation and subsequently downregulate inflammatory cytokine production. A20 is increasingly recognized as a therapeutic target for a variety of diseases, therefore, long-term exposure to low-dose LPS environmental may help promote the resolution of IAV or LPS induced inflammation, thereby improving adverse clinical outcomes both in lung tissues.

## Data availability statement

The original contributions presented in the study are included in the article/**Supplementary Material**. Further inquiries can be directed to the corresponding authors. The datasets presented in this study can be found in online repositories. The names of the repository/repositories and accession number(s) can be found at: http://proteomecentral.proteomexchange.org; PXD035611.

## Ethics statement

The animal study was reviewed and approved by the ethics committee of The College of Basic Medical Sciences of Jilin University.

## Author contributions

A-YH and FW designed and supervised the study. YG reviewed the literature and wrote the manuscript. YG, XZ, XG and ZZ performed experiments and analyzed data. SJ and ZO performed statistical analysis. A-YH revised the manuscript. FW obtained funding. All authors contributed to the article and approved the submitted version.
