# Supplementary material for: Chronic exposure to low-level lipopolysaccharide dampens influenza-mediated inflammatory response via A20 and PPAR network
Source: Front Immunol. 2023 Jan 16;14:1119473. doi: 10.3389/fimmu.2023.1119473 (PMC9886269; doi:10.3389/fimmu.2023.1119473)
Supplement: Supplementary file 1 [file DataSheet_1.docx]

Supplementary Materials for

Chronic exposure to low-level lipopolysaccharide dampens influenza-mediated inflammatory response *via* A20 and PPAR network


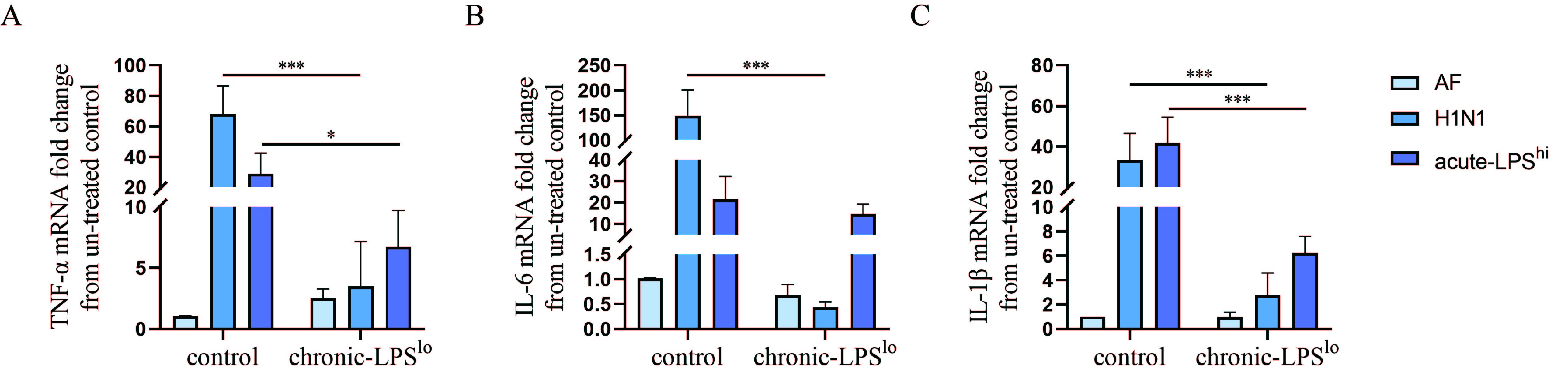


Figure S1. Effect of chronic low-dose LPS stimulation on NF-κB signaling pathway, NLRP3 inflammasome and A20 in high dose LPS-stimulated or H1N1-infected mice.

(A) TNF-α mRNA in lung tissues were detected by qPCR. (B) IL-6 mRNA in lung tissues were detected by qPCR. (C) IL-1β mRNA in lung tissues were detected by qPCR. Data are mean ± SD, n = 3.


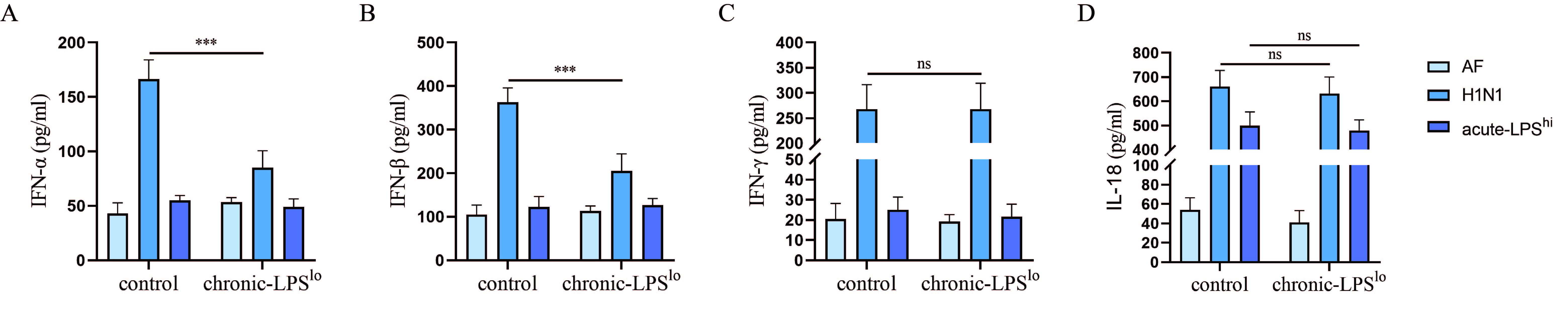


Figure S2. Effect of chronic low-dose LPS stimulation on IFN-α, IFN-β, IFN-γ and IL-18 in high dose LPS-stimulated or H1N1-infected mice.

(A) IFN-α in mouse alveolar lavage fluid were detected by ELISA. (B) IFN-β in mouse alveolar lavage fluid were detected by ELISA. (C) IFN-γ in mouse alveolar lavage fluid were detected by ELISA. (D) IL-18 in mouse alveolar lavage fluid were detected by ELISA. Data are mean ± SD, n = 3.


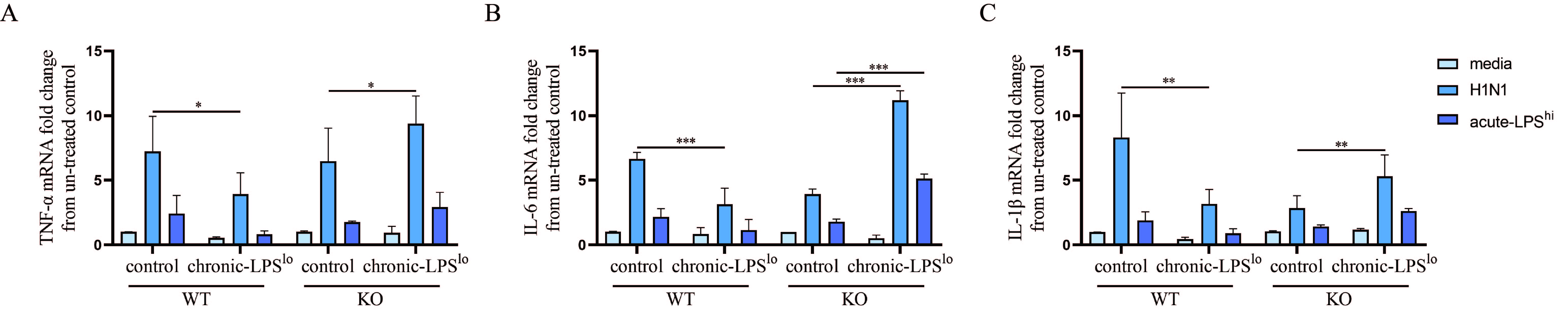
 Figure S3. Effect of chronic low-dose LPS stimulation on NF-κB signaling pathway, NLRP3 inflammasome and A20 in high dose LPS-stimulated or H1N1-infected A549^WT^ and A549^A20-KO^ cells.

(A) TNF-α mRNA in A549^WT^ and A549^A20-KO^ cells were detected by qPCR. (B) IL-6 mRNA in A549^WT^ and A549^A20-KO^ cells were detected by qPCR. (C) IL-1β mRNA in A549^WT^ and A549^A20-KO^ cells were detected by qPCR. Data are mean ± SD, n = 3.
